# Comprehensive biomechanical analysis of three clinically used fixation constructs for posterior malleolar fractures using cadaveric and finite element analysis

**DOI:** 10.1038/s41598-020-75819-7

**Published:** 2020-10-29

**Authors:** Adeel Anwar, Zhenwei Hu, Atif Adnan, Yanming Gao, Bing Li, Muhammad Umar Nazir, Cong Tian, Yanfeng Wang, Decheng Lv, Zhi Zhao, Zhen Zhang, Hu Zhang, Changgui Tong, Gang Lv

**Affiliations:** 1 Institute of Translational Medicine, China Medical University, No. 77 Puhe Road, North New Area, Shenyang, 110122 Liaoning People’s Republic of China; 2 Department of Orthopaedic Surgery, The Second Hospital of Chaoyang City, No 26, Secttion 4 Chaoyang street, Chaoyang, Liaoning People’s Republic of China; 3 Department of Human Anatomy, School of Basic Medical Science, China Medical University, No. 77 Puhe Road, 110122 North New Area, Shenyang, Liaoning People’s Republic of China; 4 Department of Orthopaedic Surgery, The Second Affiliated Hospital of Dalian Medical University, 456 Zhong Shan Road, Dalian, 116027 Liaoning People’s Republic of China; 5 Engineering Research Center of Continuous Extrusion, Ministry of Education, Dalian Jiaotong University, 794 Yellow River Road, Dalian, 116028 Liaoning People’s Republic of China; 6 Department of Anesthesia, The Second Affiliated Hospital of Dalian Medical University, 456 Zhong Shan Road, Dalian, 116027 Liaoning People’s Republic of China; 7 Department of Railway Vehicle, Ji Lin Railway Technology College, 1 Ji Hua East road, 132200 Ji Lin, People’s Republic of China; 8 Department of Orthopaedic Surgery, The First Affiliated Hospital of China Medical University, 155 Nanjing street, Shenyang, 110001 Liaoning People’s Republic of China; 9 Department of Orthopaedic Surgery, The First Affiliated Hospital of Dalian Medical University, 222 Zhong shan road, Dalian, Liaoning 116011 People’s Republic of China; 10 Department of Orthopaedic Surgery, The 920Th Hospital of Joint Logistics Support Force, Kunming, 650032 Yunnan People’s Republic of China

**Keywords:** Fracture repair, Health care

## Abstract

Different fixation modalities are available for fixation of posterior malleolar fractures (PMFs), but the best method is still unclear. The purpose of this study was to carry out a comparative biomechanical analysis of three commonly used fixation constructs for PMFs using experimental and finite element analysis (FEA). 15 human cadaveric ankle specimens were randomly divided into three groups. Specimens in group-A were fixed with two anteroposterior (AP) lag screws, group-B with two posteroanterior (PA) lag screws, and for group-C, a posterior plate was used. Each model was subjected to axial load. Outcomes included loads for 0.5 mm, 1 mm, 1.5 mm, and 2 mm vertical displacements of posterior fragments were noted. 3D FE models were reconstructed from computed tomography (CT) images and subjected to vertical loads. The model’s stress, fracture step-off, and resultant strains in implants were also studied in 3D FE models. Significantly higher amounts of mean compressive loads were observed to cause the same amount of vertical displacements in plate group (265 ± 60.21 N, 796 ± 57.27 N, 901.18 ± 8.88 N, 977.26 ± 13.04 N) than AP (102.7 ± 16.78 N, 169.5 ± 19.91 N, 225.32 ± 15.92 N, 269.32 ± 17.29 N) and PA (199.88 ± 31.43 N, 362.80 ± 28.46 N, 431.3 ± 28.12 N, 541.86 ± 36.05 N) lag screws respectively (P < 0.05). Simulated micro-motion analysis demonstrated that fracture step-off values in plate group (0.03 ± 0.001 mm, 0.06 ± 0.003 mm and 0.13 ± 0.010 mm) were the lowest among the three groups (P < 0.001). The cancellous bone showed the highest amount of stress in AP and PA lag groups respectively, whereas the lowest stress was noted in the plate-group. This biomechanical study concluded that posterior plating is biomechanically the most stable fixation construct for PMFs fixation. AP and PA lag screws with higher bone stress and fracture step-off values have a high tendency of bone cut-through and loss of fixation respectively.

## Introduction

The rotational ankle fractures are frequent in orthopaedic trauma. The occurrence of posterior malleolar fractures (PMF) is 7–44% among the ankle fractures^1–5^. Recently more attention has been paid to it. The posterior malleolar fractures are diverse in morphology and can occur in the context of rotational tri-malleolar fractures or in high energy pilon fractures as posterior variant^6–8^. Some rare cases also have been reported with isolated posterior malleolar fractures^9^. In orthopaedic literature, operative treatment of the posterior malleolar fracture remains an area of debate. Most of the authors consider the size of the posterior malleolar fracture as the decision-making parameter. They recommend the surgical fixation of fracture if fracture size accounts for more than 25% of tibial plafond^10,11^. Clinically, the posterior malleolar fractures can be approached either using the indirect anterior approach with subcutaneous anterior to posterior (AP) lag screws fixation or through a direct posterolateral or posteromedial approach in which reduction is maintained either by posteroanterior (PA) lag screws or a posterior buttress plate fixation. Unsatisfactory functional outcomes are reported in unstable ankle fractures with the involvement of posterior malleolus^1,12^. Though most of the surgeons recommend the reduction of posterior malleolus using the direct posterior approaches, still there is an ongoing debate about the optimal type of fixation used for posterior malleolar fractures^4,13–18^. O’Connor and colleagues in a clinical study encouraged more promising results on follow up with posterolateral plating as compared to anteroposterior lag screws^18^. However, according to another study, there was no significant difference irrespective of the type of fixation used for posterior malleolar fixation^19^. Trauma surgeons need to understand the comparative biomechanical efficacy of different fixation methods; so that they can select optimal fixation construct for better clinical outcomes. The previous biomechanical studies had compared only two fixation methods, i.e., AP screws vs. plate and PA lag screw vs. posterior plate^20,21^. There is still a need for a study in which we can use the cadaveric specimens and finite element analysis simultaneously to address this issue. So in this study, we have conducted a comprehensive biomechanical analysis for the fixation of > 25% sized posterior malleolar fractures using three different fixation modalities. The cadaveric specimens were used to conduct a biomechanical comparison between three fixation methods (AP, PA lag screws, and posterior plating). Additionally, a finite element analysis was also performed to evaluate the precise picture of micro-stress changes in boney components. This might be an indicator of cut-through stress changes in the cancellous bone caused by fixation implants, especially the lag screws. The strain in fixation implants was also studied.

## Materials and methods

### Ethics and informed consent

This research work was approved by the Medical Research Ethics Committee of our institution (Approval no: 2017-KB-48) and was performed in accordance with institutional rules and regulations. All the cadaveric specimens used in this research work were collected from the organ donation center of our institution. None of the tissue donors were from a vulnerable population, and all donors or next of kin provided written informed consent that was freely given. For CT scans used in this study, the affiliated hospital has granted ethical approval. The participants gave free informed consent about X-rays and CT scans.

### Preparation of specimens

**Figure 1:**
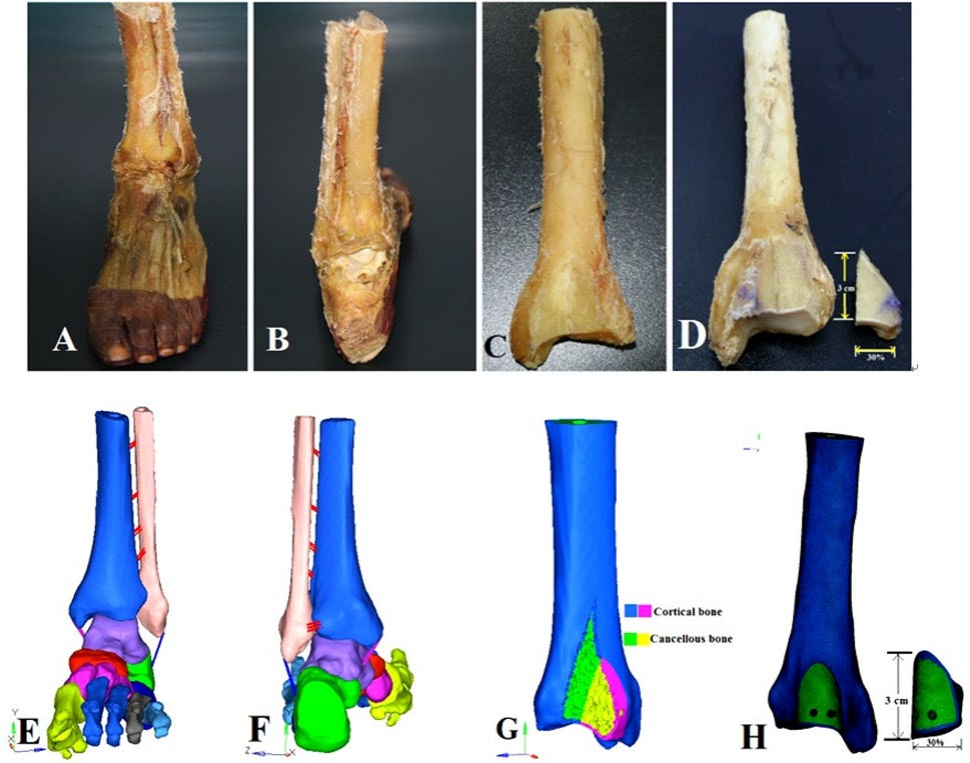
Cadaveric specimens and validated 3D fracture models. (**A**) intact specimen anterior view, (**B**) posterior view, (**C**) section showing cortical and cancellous portions, (**D**) distal tibia showing fracture fragment with vertical length “L” of 3 cm and percentage of the articular surface (30%). (**E**–**H**) representing corresponding 3D models.

In total, fifteen human cadaveric frozen specimens of the mid tibia to toe-tip length were used in this study. The specimens consisted of 11 tibiae of males and 4 tibiae of females, with the mean age of 55 years (range: 50–60 years) and mean body weight of 80 kg (79.75 ± 6.88). All the specimens were thawed at the room temperature 24 h before use (Fig. 1A,B showing initial specimens before handling with corresponding finite element model in Fig. 1E,F). The previous study using the combined bony ligamentous ankle specimens has shown that the biomechanical-testing failures happened at the place other than the posterior malleolus^20^. Subsequently, the fibulae and other soft tissue structures were removed before the biomechanical evaluation (see Fig. 1C,D). The size of simulated fracture fragments used in this study (30% of the articular surface) was based on the average fracture size calculated from the 20 patients with posterior malleolar fractures treated at the author’s hospital. The morphological parameters of posterior malleolar fractures are summarized in Table 1.

**Table 1:** Numerical data obtained from CT scanning of 20 patients with posterior malleolar fractures.

S. No	Length of fragment (cm)	% of plafond involved
1	3.08	20.76
2	3.88	29.82
3	3.10	34.51
4	3.16	34.90
5	4.10	35.19
6	3.41	26.92
7	2.71	26.97
8	3.09	26.19
9	3.77	28.48
10	3.87	29.32
11	3.90	49.21
12	3.46	27.44
13	2.87	26.43
14	3.27	25.47
15	2.51	35.54
16	2.93	23.70
17	3.46	24.34
18	3.09	39.63
19	3.34	24.36
20	2.24	24.78
**Mean**	3.26 (2.24 to 4.10)	29.69 (20.76 to 49.21)
**SD**	0.49	6.70

The fragment-size measurements were marked with ink and then a thin blade power saw was used to make the osteotomy cuts. The dividing fracture plane was 50 degrees to 60 degrees with the joint surface. On the other hand, this dividing plane was extended to the posterior metaphysis of the tibia. The resulted fracture fragment involved 30% of the tibial plafond (Fig. 1D). Though there is no consensus about the surgical fixation standards, most of the surgeons regard > 25% sized fragment as an indication for surgical fixation. Thus, the fracture model used in this study has fulfilled the so-called reference value that is believed to be addressed surgically according to the recent literature^10,12,22^.

### Grouping of models

**Figure 2:**
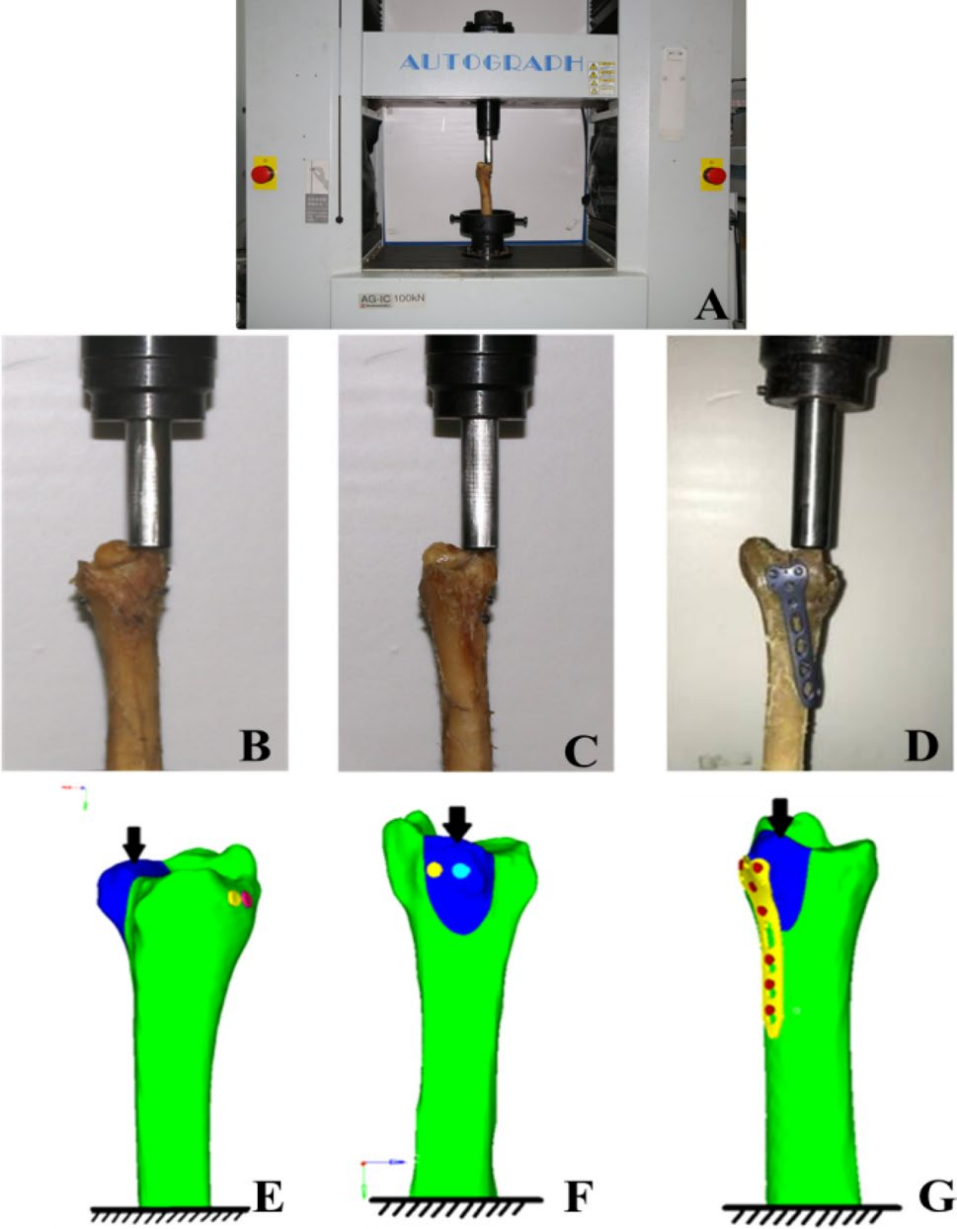
Experimental setup and different fixation methods: (**A**) Material testing machine (Autograph-AGIC). Cadaveric specimens showing fixation with (**B**) AP lag screws, (**C**) PA lag screws, and (**D**) posterior plate. (**E**–**G**); depict corresponding 3D simulation models.

All of the specimens were reduced and fixed by a single resident orthopaedic surgeon under direct vision. The posterior malleolar fracture models were anatomically reduced and initially fixed with two K-wires. Finally, the random fixation was achieved using the lag screws and plates in the three groups as; Group A with two inter-fragmentary 3.5 mm fully threaded cortical lag screws placed in anteroposterior (AP) direction, Group B with two 3.5 mm posteroanterior (PA) lag screws. AP and PA screws were secured using the lag-technique to compress the fracture. In Group C, a posterior malleolar anatomic plate was used, as shown in Fig. 2B–D).

### Biomechanical testing

The ankle joint undergoes two to five times the load of the body weight, and the articular surface of the posterior malleolus bears nearly 25% of this load^23–25^. The mean body weight was 80 kg (800 N) in this study. Considering the 5 times of this weight as a reference value, a total of 4000 N was the highest value of load in Newton (N) supposed to be taken by the articular surface of the distal tibia. Therefore, 1000 N (25%) load was shared by the posterior malleolus. Each specimen was fixed vertically at the base of the Material Testing Machine (Shimadzu Autograph AGIC 5569, Japan) using a custom-made fixation stand (Fig. 2A). A pilot study was conducted before this study to determine the load scale for the specimen’s size, speed of the loader, the loader’s exact position, and the specimens and implant failure load. A vertical compressive load with a 1 mm/min speed was applied to the posterior malleolar fragments in all models. The failure load was assumed the amount of applied force that produced 2 mm displacement^26^. According to our observation, the full thickness of the articular cartilage was nearly 1 mm in the anatomical specimens. In previous research, Drijfhout et al. stated that there were higher rates of degenerative osteoarthritis in individuals with post-operative step-off of 1 mm or more^27^. That’s why we take 1 mm displacement as the reference value in this study. For comprehensive biomechanical analysis, the amount of deforming loads for the vertical subsidence of posterior fragment for 0.5 mm, 1.0 mm, 1.5 mm, and 2.0 mm were noted. All the models were subjected to the failure load after calculating the individual implant’s fixation strength.

### Finite element simulation study

#### 3D modeling and validation of finite element model

A total of 225 CT scan DICOM images of a volunteer were imported into Mimics software (version 10.1, Materialise, Leuven, Belgium) (https://www.materialise.com) to reconstruct the 3D surface geometry of distal tibia, fibula and foot bones by using the region-growing method. There was no history of ankle trauma or degenerative disorders. The surface masks were subsequently edited to smooth the 3D models. Then bones structures in IGS files were imported into Geomagics Studio software (version 11.0, Raindrop Company, USA) (https://www.geomagic.com). Here volumes of the bones were obtained and then exported as STP files for Solid Works 2012 (DS Solid Works Corp., USA) (https://www.solidworks.com) processing and modeling. Finally, the separate solid objects representing the bones were assembled within Solid Works to get a 3D foot–ankle complex (Fig. 1E,F). To simulate posterior malleolar fracture, the posterolateral margin of the distal tibia was modeled according to the data in Table 1 (30% fragment size) (see Fig. 1G,H). To establish the exact fit fracture model, there was no fracture gap between the simulated fragments. This was done in accordance with the experimental study to represents the ideal anatomic reduction. However, the posterior fragment can move relative to the un-fractured tibia. Three different fixation modalities (AP lag screws, PA lag screws, and the posterior plate) were adapted to fix all the fracture models, as shown in Fig. 2E–G. The 3D models of the implants were designed using computer-aided design (CAD) software (Solid Works 2012, DS Solid Works Corp., USA) (https://www.solidworks.com). Each model was meshed using HyperMesh (version 11.0, Altair Engineering, Inc, USA) (https://www.altair.com). The cortical and cancellous portions of the tibia were separated in Hyper Mesh during the meshing process. The 2D auto-mesh (2D shell) was initially used for the cortical bone, and tetrahedral C3D10 elements (3D tetra-mesh) were used to mesh the cancellous portion^28^. The 3D tetra-meshing was followed by organizing the elements into a new folder by selecting the elements (by face) and then consecutively by adjacent and finally move the selected elements to a new folder (3D cortical bone mesh). Finally, the quadratic tetrahedral mesh was obtained in cortical and cancellous parts. The cortical and cancellous parts are shown in Fig. 1G. Based on the mesh convergence study, the mesh size of 1 mm was selected. Note that the threads of the screws were simplified with little impact on the results according to previous studies^29,30^. The types of interaction in the simulation were adopted for real biomechanics. There were 7 interactions in each lag screw model (group A and group B), and 16 in the plate fixation model. To mimic the mechanical nature of the lag screw, the tie-interaction was used between the screw (head of the screw and distal 1/3rd part) and the bone. The screw shaft was set to slide against the bone surface using surface-to-surface contact. For the plate model, the tie-interaction was used between plate and screws as well as between screws and bone to demonstrate the locking plate mechanics. The friction coefficient for bone-bone interactions was assigned to 0.46, 0.3 for bone-implant interactions and 0.23 for implant-implant interactions^31^. Validation of 3D models was done with the previous literature^30–36^.

#### Material properties and boundary conditions

The reconstructed 3D boney models (fractured tibia and fixation construct) in the inp form were then imported into finite element analysis software Abaqus (version 6.14, Simulia Corp., USA) (https://www.simulia.com). The bones were assumed to behave as homogeneous, isotropic, and linearly elastic material^30–34^. The cortical and cancellous portions of the distal tibia were modeled with Young’s modulus of 7300 and 1100 MPa and the Poisson ratio of 0.3 and 0.26 respectively^35^. Fixation implants, including screws and plate, were assigned an elastic modulus and the Poisson’s ratio of 110,000 MPa and 0.3 respectively^36^. The effect of gravity was considered negligible in the models. The proximal end of the tibia was fixed in all degrees of freedom, whereas force was applied to the fracture fragment, as shown in Fig. 2E–G. Three different magnitudes of axial force 400 N, 800 N, and 1600 N representing half of the body-weight, one body-weight and two times of the body weight were simulated respectively.

### Statistical analysis

Statistical analysis was done using SPSS 16.0 (SPSS Inc., Chicago, IL). The parametric statistics were conducted to perform One-way ANOVA and multiple comparisons Least-Significant Difference (LSD), post hoc tests to determine the *P-*values for vertical displacement and failure loads. *P*-value was considered significant when *P* < 0.05.

### Ethics approval

Ethics approval was obtained before this study.

### Consent to participate

Written informed consent was obtained and all the participants were given full consent freely.

### Consent for publication

All the participants were agreed and were given written informed consent for publication freely.

## Results

### Vertical displacement

**Figure 3:**
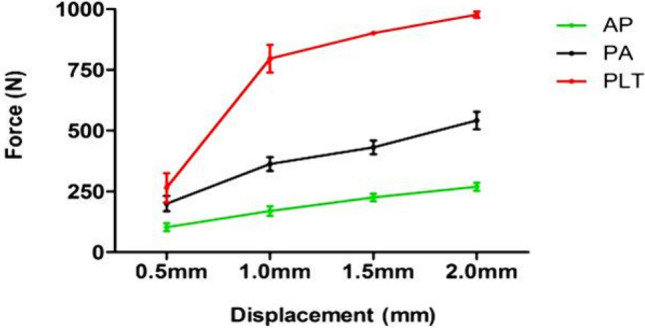
Graphical representation of vertical displacements (cadaveric study) and the applied forces in three groups.

**Figure 4:**
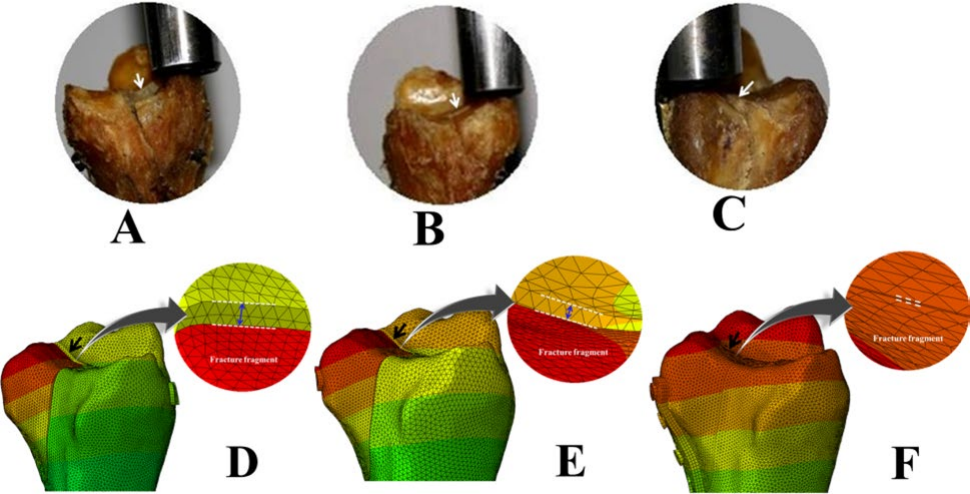
Cadaveric specimens and 3D models showing vertical step-off in (**A**) AP lag screws, (**B**) PA lag screws, (**C**) Post. plate, (**D**–**F**) corresponding simulation results.

The vertical displacement values (mm) of the posterior fragment and the corresponding axial loads (N) in the three groups are summarized in Table 2. The mean axial loads to cause 0.5 mm, 1.0 mm, 1.5 mm, and 2.0 mm vertical displacements of the posterior fracture fragment in AP lag (group A) were significantly lower than the group B and group C (*P* = 0.000) (see Figs. 3 and 4A–C; white arrow for anatomic specimens, Fig. 4D–F; black arrow for 3D models). It signifies the lowest biomechanical stability by using the AP lag fixation. On the other hand, the comparison between the posterior plate (group C) and PA lag screws showed significant strength of the posterior plate at 0.5 mm (P < 0.05) and 1.0 mm, 1.5 mm, and 2.0 mm displacements (P < 0.001) respectively.

**Table 2:** The amount of applied loads (N) for 0.5 mm, 1 mm, 1.5 mm, and 2 mm vertical displacements and the failure load of the different groups (cadaveric study).

Groups	Vertical step off (mm)				
		0.5 mm	1.0 mm	1.5 mm	2.0 mm
A (AP lag)	Mean axial loads (N) Mean ± SD	102.70 ± 16.78	169.50 ± 19.91	225.32 ± 15.92	269.32 ± 17.29
B (PA lag)		199.88 ± 31.43	362.80 ± 28.46	431.30 ± 28.12	541.86 ± 36.05
C(Post plate)		265.00 ± 60.21	796.00 ± 57.27	901.18 ± 8.88	977.26 ± 13.04
Mean difference*	A–B	− 97.18	− 193.30	− 205.98	− 272.54
	B–C	− 65.12	− 433.20	− 469.88	− 435.40
	C–A	162.30	626.50	675.86	707.94
*P*-value		0.003^A-B^	0.000^A-B^	0.000^A-B^	0.000^A-B^
		0.026^B-C^	0.000^B-C^	0.000^B-C^	0.000^B-C^
		0.000^A-C^	0.000^A-C^	0.000^A-C^	0.000^A-C^

*The mean difference is significant at *P* < 0.05, ^A^ Group A (AP lag), ^B^ Group B (PA lag), ^C^ Group C (Post plate).

### Stress patterns/cut through bone

**Figure 5:**
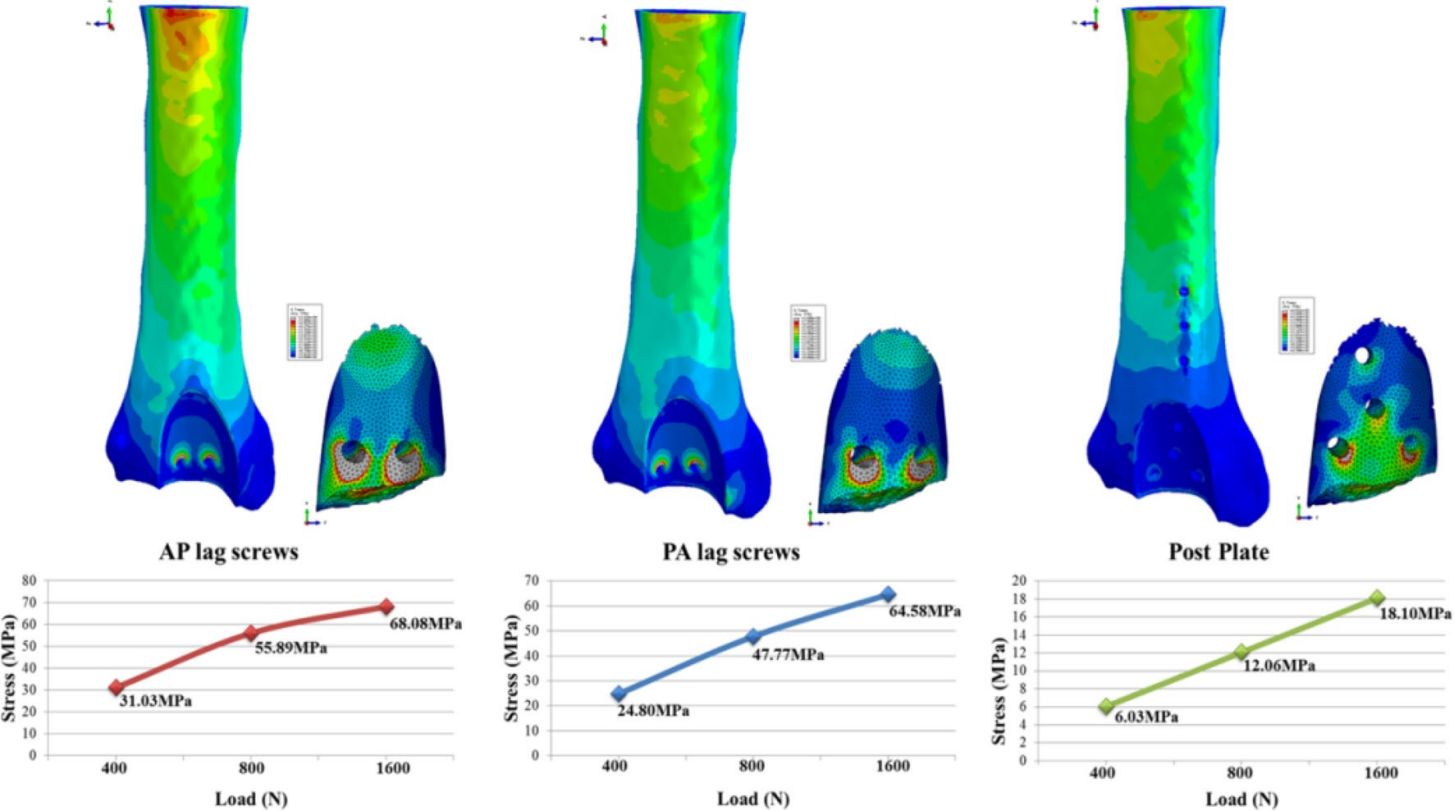
Tresca stresses in the fracture fragments fixed with different constructs.

Mainly the Tresca stress patterns were concentrated in lower screw holes of all models. The highest Tresca stress was noted in the AP lag model (31.03 MPa, 55.89 MPa, 68.08 MPa). PA lag model showed relatively lower stress (24.80 MPa, 47.77 MPa, 64.58 MPa), whereas in the posterior plate, the lowest stress (6.03 MPa, 12.06 MPa, 18.10 MPa) was noted in the cancellous portion of the fracture fragment. In all the models, maximum stress regions are around the holes of screws, and in AP and PA lag groups, these regions might have cut through the cancellous portions of bone. Tresca stress distributions in the three models are shown in Fig. 5.

### Fracture step-off (micro-motion analysis)

The fracture micro-motion analysis demonstrated that the fragment vertical step-off in the plate group (0.03 ± 0.001 mm, 0.06 ± 0.003 mm, and 0.13 ± 0.010 mm) was the lowest among the three models. Whereas relative motions in PA lag models (0.26 ± 0.009 mm, 0.53 ± 0.003 mm, and 0.93 ± 0.015 mm) at all applied forces were lower than AP group (0.46 ± 0.005 mm, 0.83 ± 0.004 mm, and 1.64 ± 0.020 mm). When the magnitude of the applied load increased, the step-off in the individual group was also increased (see Fig. 4D–F). The statistical analysis of nodal relative displacement is given in Table 3.

**Table 3:** The nodal statistical analysis of vertical step-off of fracture fragment in 3D models.

Groups	Applied loads (Mean ± SD)			
		400 N	800 N	1600 N
AP	Micro-motion/ Step-off of fragment	0.46 ± 0.005 (0.4449 to 0.4684)^#^	0.83 ± 0.004 (0.8213 to 0.8414)^#^	1.64 ± 0.020 (1.5903 to 1.6897)^#^
PA		0.26 ± 0.009 (0.2373 to 0.2834)^#^	0.53 ± 0.003 (0.5265 to 0.5415)^#^	0.93 ± 0.015 (0.8887 to 0.9646)^#^
Plate		0.03 ± 0.001 (0.0285 to 0.0335)^#^	0.06 ± 0.003 (0.0532 to 0.0664)^#^	0.13 ± 0.010 (0.1052 to 0.1548)^#^
*P*-value*		0.000^A-B^	0.000 ^A-B^	0.000 ^A-B^
		0.000 ^B-C^	0.000 ^A-B^	0.000 ^A-B^
		0.000^A-C^	0.000^A-C^	0.000^A-C^

*Significant at *P* < 0.05, ^#^95% Confidence Interval for Mean.

### Strain analysis of fixation implants

**Figure 6:**
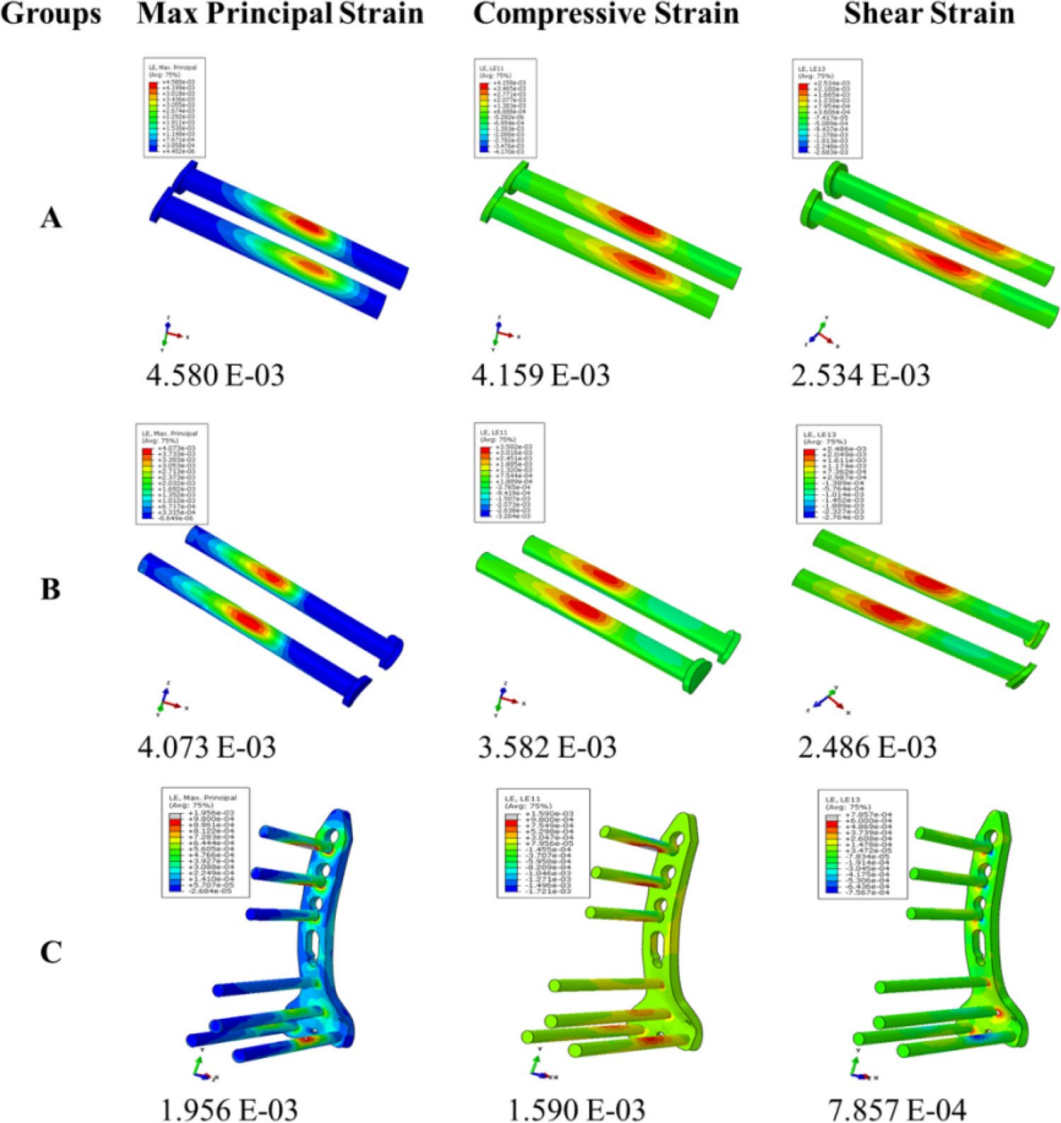
Strain analysis of individual implants in three groups by using 400 N load.

**Figure 7:**
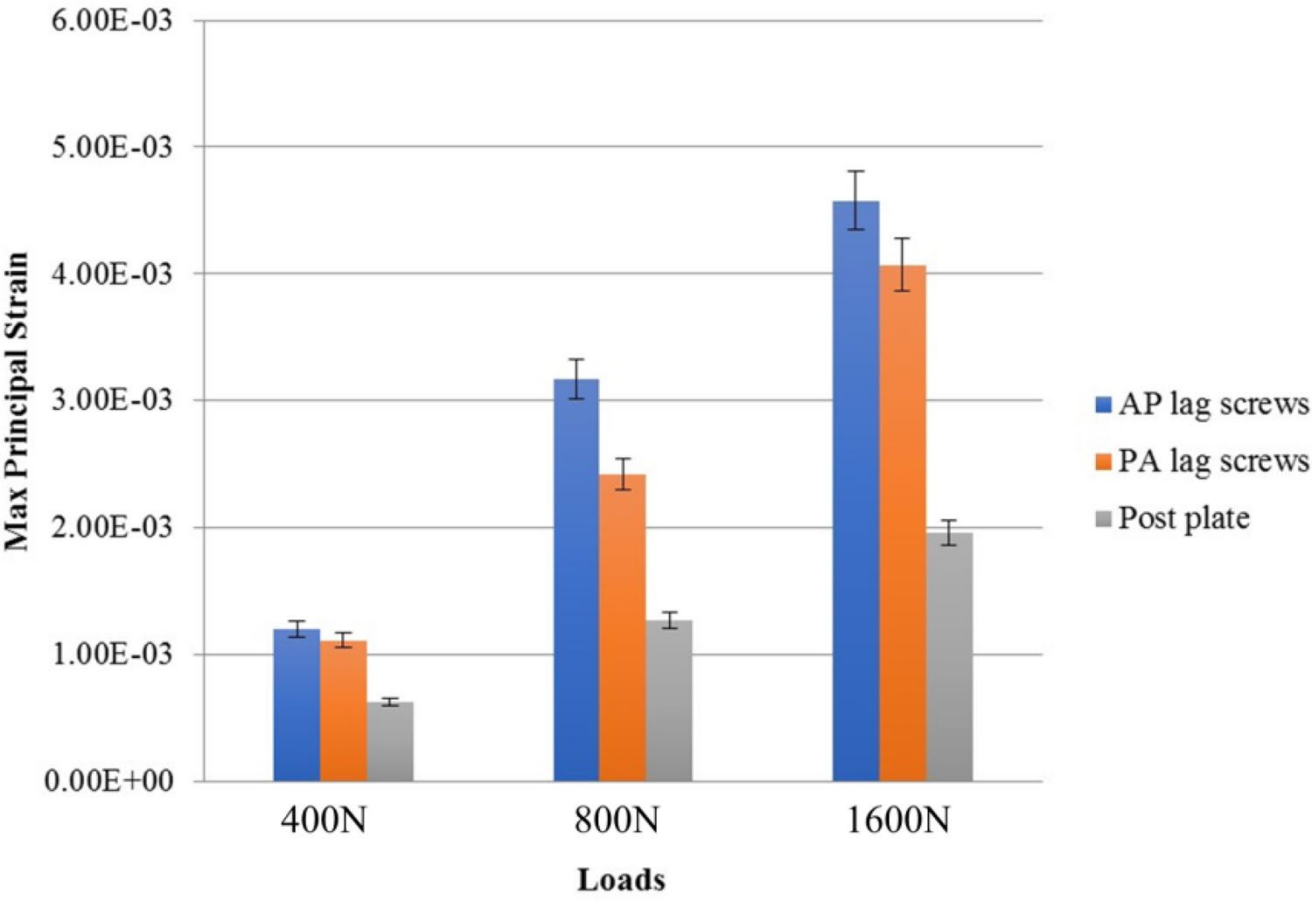
Comparative analysis of Max principal strain in fixation implants using different applied loads.

The posterior plate group showed the lowest (1.956E−03) value of the principal strain than group B (4.073E-03) and group A (4.580E-03). The compressive and shear components of strain were higher in lag screw groups (group A and group B) and lowest in the plate group. The detailed strain analysis of the three groups on applying 400 N load is shown in Fig. 6A–C. In lag screws, the strain was mainly concentrated near the fracture line areas. On the other hand, in the plate group, the principal strain and compression strains were uniformly distributed in all screws. The shear strain was dominated in regions of screws and plate junction. The principal strain in three fixation groups using 400 N, 800 N, and 1600 N loads is given in Fig. 7.

## Discussion

Achieving the congruent and stable joint surface is the key predictor for positive outcomes in the ankle joint fractures. Biomechanically, the posterior malleolus plays a critical role in tibiotalar load transfer and resists posterior talar subluxation^37^. The posterior malleolus also works as an anchorage site for the posterior inferior tibiofibular ligament. Therefore, anatomic fracture reduction and stable fixation also contribute to syndesmotic strength and stability^38^. In modern orthopaedic practice; active exercises are encouraged to prevent possible joint stiffness and to promote early return to daily activities. For this reason, the fixation construct should be stronger enough to hold the fracture fragment in a reduced position during the healing process. Less stable fixation may result in reduction loss and a successive increase in the focal contact pressure in tibial plafond, leading to degenerative arthritis^10,39^. According to our findings, the mean value of axial loads that caused the 1.0 mm step-off in the posterior plate group (796.00 ± 57.27 N) was more significant than the mean values for the PA (362.80 ± 28.46 N) and AP (169.50 ± 19.91 N) lag screws. Hence, the posterior plate resisted the vertical subsidence more effectively, and a greater amount of load was required to move the fixed fracture fragment as compared to AP and PA groups. The load for implant failure (2.0 mm subsidence) was also higher in the posterior plate group (977.26 ± 13.04 N) than group B (541.86 ± 36.05 N) and group A (269.32 ± 17.29 N). These findings are in accordance with previous biomechanical and clinical studies^18,20,21^. In a biomechanical study, Bennett et al. concluded that the posterior buttress plating showed less displacement than AP lag screws^20^. A study published in Chinese literature documented that the posterior malleolus fixation with a distal radius plate was more stable than the PA lag screws^21^. The improved clinical outcomes were noted at follow up in patients treated with plate compared to AP screws^18^. We also noted that the mean load for 2.0 mm vertical displacement in group B (PA lag screws) was higher than group A (AP lag screws) with a mean difference of 272.54, so we can conclude that PA lag screws are biomechanical more stable than AP lag screws. Therefore, we advocate that when there is a choice between AP and PA lag screws only, it is better to select PA lag screws than AP lag due to greater stability offered by the same screws but in the opposite direction (i.e., from posterior to anterior direction).

**Figure 8:**
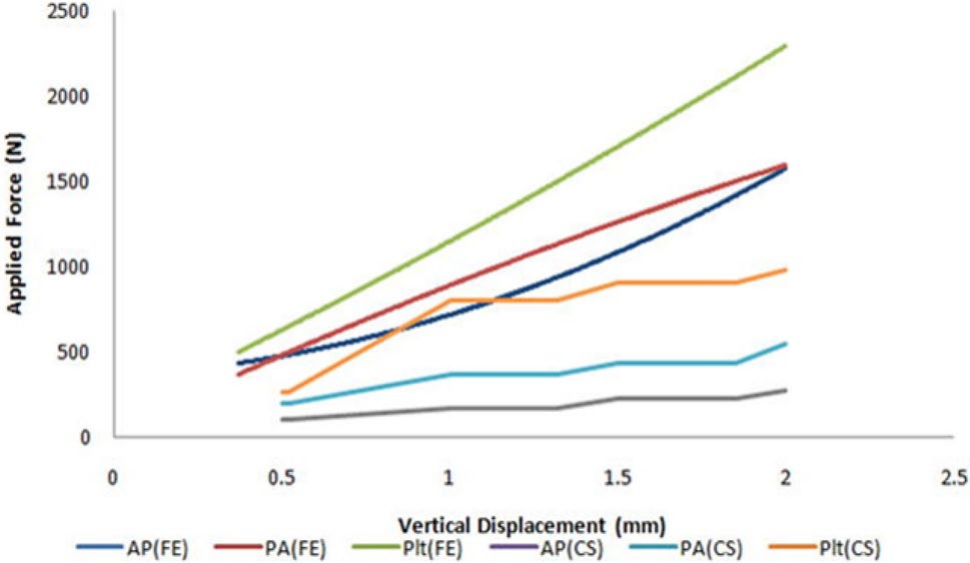
Graphical representation of a comparison between cadaveric and simulation study.

Moreover, the direct posterior approach also provides posterior cortical-read, which helps to ensure the reduction quality. Results of the simulation study concluded that the posterior plate was the most stable fixation method than AP and PA lag screws because it has the lowest vertical step-off (relative fracture displacement) values as represented by an arrow in Fig. 4D–F. By increasing the applied load, there was nearly two folds increase in the relative vertical displacement (Table 3). The significant Tresca stress values in AP and PA lag groups can cause cut-through in cancellous bone. In these fixation methods (lag screws), the earlier weight-bearing has a greater risk of bone destruction and subsequent fixation failure. On the other hand, the lowest and uniform Tresca stress distribution in the posterior plate produced a bone-protection effect. The plate has the advantage of dispersing the strain across the implant (Fig. 6). The principal strain was mostly concentrated in the regions of screws-plate interaction in the proximal plate and the screws near the plate-screws interactions. The shear and compressive strains (7.857E−04, 1.590E−03) were lowest as compared to group A (2.534E−03, 4.159E−03) and group B (2.486E−03, 3.582E−03). In lag screws, the strain was concentrated in screws near the fracture line. The higher shear strain values in both lag groups increase the risks of fatigue failure. In Fig. 8, it is clear that in the simulation study, the curves are nearly straight, but in the case of the experimental study, these are not straight. It can be explained by the reason that in the experimental study, the fracture surface is rough (actual condition), and for simulation, it was smooth. But irrespective of this, the final results (in both conditions) showed that the posterior plate was biomechanically most stable, and AP lag screws were the least stable at the given conditions.

This work still has some limitations. First of all, in a real clinical situation, there are soft tissues, including the ligaments, but in this study, we removed all the soft tissues from the specimens, and 3D models were also established accordingly. So only the implant’s strength to resist the vertical subsidence was tested. But this limitation was applied in all the groups equally, so it did not interfere with the final findings' validity. Another reason for this was that it was necessary to dissect the soft tissues in anatomic specimens to get fracture fragment models of exact size and parameters. Also, the previous study dictated that by using the combined bony-ligamentous ankle specimens showed testing failures happened at the place other than the posterior malleolus^20^. Moreover, in this study, we have reduced the fracture fragments under direct vision, but clinically it may be challenging to attain the perfect anatomic reduction in every case.

## Conclusions

Comparing the cadaveric and FE simulation study, we have concluded that posterior plating with the highest resistance to vertical displacement is biomechanically the most stable fixation method for fixation of posterior malleolar fractures involving 30% articular surface. The second most stable construct is PA lag screws, whereas the AP lag group with the lowest value of deforming loads to cause the same amount of vertical displacements is the least stable fixation method among the three groups. The lag screws in AP and PA directions with higher bone stress and relative fracture displacement (step-off) values have a high tendency of bone cut through and loss of fixation, respectively. Surgeons should consider the findings of this biomechanical study when the decision is made about the selection of optimal fixation constructs for the posterior malleolar fractures with > 25% involvement of articular surface.
